# Variation in Drug Sensitivity of Malignant Mesothelioma Cell Lines with Substantial Effects of Selenite and Bortezomib, Highlights Need for Individualized Therapy

**DOI:** 10.1371/journal.pone.0065903

**Published:** 2013-06-20

**Authors:** Adam Szulkin, Gustav Nilsonne, Filip Mundt, Agata M. Wasik, Pega Souri, Anders Hjerpe, Katalin Dobra

**Affiliations:** 1 Karolinska Institutet, Department of Laboratory Medicine, Division of Pathology, Stockholm, Sweden; 2 Karolinska Institutet, Department of Clinical Neuroscience, Stockholm, Sweden; 3 Stockholm University, Stress Research Institute, Stockholm, Sweden; IIT Research Institute, United States of America

## Abstract

**Background:**

Malignant mesothelioma cells have an epithelioid or sarcomatoid morphology, both of which may be present in the same tumor. The sarcomatoid phenotype is associated with worse prognosis and heterogeneity of mesothelioma cells may contribute to therapy resistance, which is often seen in mesothelioma. This study aimed to investigate differences in sensitivity between mesothelioma cell lines to anti-cancer drugs. We studied two novel drugs, selenite and bortezomib and compared their effect to four conventional drugs. We also investigated the immunoreactivity of potential predictive markers for drug sensitivity; Pgp, MRP-1, ERCC1, RRM1, TS, xCT and proteasome 20S subunit.

**Materials and methods:**

We treated six mesothelioma cell lines with selenite, bortezomib, carboplatin, pemetrexed, doxorubicin or gemcitabine as single agents and in combinations. Viability was measured after 24 and 48 hours. Immunocytochemistry was used to detect predictive markers.

**Results:**

As a single agent, selenite was effective on four out of six cell lines, and in combination with bortezomib yielded the greatest response in the studied mesothelioma cell lines. Cells with an epithelioid phenotype were generally more sensitive to the different drugs than the sarcomatoid cells. Extensive S-phase arrest was seen in pemetrexed-sensitive cell lines. MRP-1 predicted sensitivity of cell lines to treatment with carboplatin and xCT predicted pemetrexed effect.

**Conclusions:**

The observed heterogeneity in sensitivity of mesothelioma cell lines with different morphology highlights the need for more individualized therapy, requiring development of methods to predict drug sensitivity of individual tumors. Selenite and bortezomib showed a superior effect compared to conventional drugs, motivating clinical testing of these agents as future treatment regime components for patients with malignant mesothelioma.

## Introduction

Malignant mesothelioma (MM) is a therapy resistant tumor, originating from mesothelial cells covering the serous cavities of the pleura, pericardium or peritoneum [1], [2]. The tumor is associated with exposure to asbestos and appears most often in the pleura [2], [3]. Mesothelioma cells are classified as being either epithelioid or sarcomatoid. Hence, three different histopathological appearances are possible; one dominated by the epithelioid phenotype, one dominated by the sarcomatoid phenotype and one biphasic type including cells of both phenotypes [2], [4].

Several studies have demonstrated differences in gene-expression between the two phenotypes [5], [6], [7], [8], and identified various components of the proteasome and redox systems as potential therapeutic targets. Our previous studies have indicated a phenotype-dependent sensitivity to experimental drugs or chemotherapeutic agents which are known to target these systems [9], [10], [11]. Differentiation related sensitivity profiles correlate to clinical findings, and patients with a tumor dominated by the sarcomatoid phenotype accordingly have a worse prognosis [4].

Currently, standard treatment for MM combines pemetrexed and cisplatin with a 40% response rate, an average increase in survival time of 3 months and a median survival time of 1 year [1], [12], [13], [14]. Comparable results have been achieved in phase II studies using the combination of pemetrexed and carboplatin [15], as well as combining carboplatin, liposomized doxorubicin and gemcitabine [16]. We have previously reported strong phenotype-dependent effects of selenite and PSI, a proteasome inhibitor similar to bortezomib, on mesothelioma cells [9], [10], [11]. Others have shown promising results for selenite in early clinical trials in different human tumor types [17], [18].

In this study, we aimed to further evaluate the phenotypic differences in sensitivity of mesothelioma cells to experimental and conventional anti-cancer drugs. Therefore, we investigated the cytotoxicity of six drugs and their pairwise combinations on a panel of six mesothelioma cell lines of epithelioid, biphasic or sarcomatoid growth patterns. We included two experimental drugs: selenite and bortezomib. Selenite is a modulator of the redox system, and we further investigated its phenotype-dependent effect and potential synergistic effects with other drugs [10], [11]. We evaluated the effect of bortezomib, a proteasome inhibitor that has been demonstrated to be cytotoxic on mesothelioma cells [9], [19], [20]. These effects were compared to the aforementioned conventional drugs; pemetrexed, carboplatin, doxorubicin, and gemcitabine. Carboplatin was the only platinum drug included since it has been shown that cisplatin and selenite interact *in vitro* [21], and because of the demonstrated effect of carboplatin in combination with liposomized doxorubicin and gemcitabine [16].

We also investigated the immunoreactivity of seven different markers, proposed to predict drug sensitivity. Increased expression of P-glycoprotein (Pgp) correlates to an increased in vitro resistance to taxol and doxorubicin [22]. Expression of Multidrug resistance-associated protein 1 (MRP-1) correlates to doxorubicin sensitivity [23]. Expression of Excision repair cross-complementing rodent repair deficiency, complementation group 1 (ERCC1) and Ribonucleotide reductase M1 (RRM1) correlates to treatment effect of gemcitabine or carboplatin [24], [25], [26], [27]. Thymidylate synthase (TS) is the main target for pemetrexed [28] and a low expression of TS has been correlated to a higher overall survival of MM patients treated with pemetrexed and a platinum agent [29], [30]. Selenite toxicity mainly depends on the level of selenium accumulated in the cell and it has been shown in cell lines that high expression of x_c_^-^ cystine transporter (xCT) and MRP-1 causes an increased uptake of selenite [31], [32]. High expression of 20S proteasome (20S P) has been correlated to bortezomib sensitivity [33].

Our study shows that MM treatment could benefit from including drug combinations with selenite and bortezomib into standard treatment. Also, our results indicate that a greater understanding of phenotype-related cell sensitivity as well as correlation to predictive markers could lead to increased clinical drug efficacy. We therefore suggest that MM treatment should be of a personalized treatment character.

## Materials and Methods

### Cells and Cell Culture

This study was performed using six different MM cell lines. Jurkat T-cell lymphoma cells were used as controls. MM cell lines: STAV-AB, STAV-FCS and ZL-34 cells were kindly provided by Julius Klominek [34], [35]. DM-3 and JL-1 cells were obtained from the Deutsche Sammlung von Mikroorganismen und Zellkulturen (DSMZ) [36]. M-14-K cells were kindly provided by K. Linnainmaa [37] and Jurkat cells were obtained from the American Type Culture Collection (ATCC) [38]. The DM-3 and JL-1 cells were cultivated in NCTC-109 medium (Sigma-Aldrich, St. Louis, MO, USA) with 1% L-glutamine (Invitrogen, Carlsbad, CA, USA) and 20% FBS (Fetal Bovine Serum, Invitrogen). The M-14-K, ZL-34, STAV-FCS and Jurkat cells were cultured in Gibco RPMI 1640 medium with 25 mM HEPES buffer (Invitrogen) and 1% L-glutamine, 5% FBS and 5% BS (Bovine Serum, Invitrogen). The STAV-AB cells were grown in Gibco RPMI 1640 medium with 25 mM HEPES buffer and 1% L-glutamine and 10% human AB-serum. All cell lines were cultured in 37°C at 5% CO_2_ in 75 cm^2^ flasks (Sarstedt, Nümbrecht, Germany).

### Cell Line Characteristics

For morphological characterization, micrographs were randomly taken of untreated cells at 40–90% conﬂuency. To further characterize the phenotypic differences of the tumor cells, length/width ratios for the six MM cell lines were calculated by measuring the longest diameter and the perpendicular diameter at the center of the nucleus [39]. For each cell line, 100 cells from at least three different micrographs were measured. Average doubling time was calculated from growth curves of untreated cells in cytotoxicity tests.

### Cytotoxicity Test

Confluent cells were detached with 0.5% trypsin (Invitrogen) and the amount of cells was estimated by measuring the absorbance of the cell suspension using an Ultrospec 10 cell counter (Biochrom Ltd, Cambridge, UK) and related to reference curves. The cells were then centrifuged and the pellet was resuspended. Titrations of cell plating densities were performed for each cell line to ensure optimal logarithmic growth conditions. Cells were seeded in 96 well microtiter plates with 100 µl culture medium containing different drugs (diluted in PBS) or PBS for the respective controls. Cells were incubated for 24 or 48 hours and WST-1 (Water Soluble Tetrazolium-1, Roche, Mannheim, Germany) was added to measure the cytotoxicity of the different drugs and drug combinations. Mitochondrial enzymes cleave WST-1 generating a colorimetric product strictly correlated to the metabolic activity of the cell population and thus proportional to the amount of live cells. Absorbance of the colorimetric product was measured at 450 nm and normalized by subtracting background absorbance at 600 nm.

### Selection of Drug Concentrations

The drug concentrations used in this study represent the average IC_30_ values (data not shown) of the STAV-AB and STAV-FCS cells. When an IC_30_ value was not observed within the clinically relevant dose range (for pemetrexed and carboplatin) we used the highest concentration in this range. These concentrations were calculated from the maximal dose and the volume of distribution obtained from the United States Food and Drug Administration [40], [41], [42], [43], [44], [45] and others [46]. The lack of an observed IC_30_ effect using WST-1 was confirmed by the Annexin V/PI assay (data not shown), performed as previously described [10]. Consequently, doxorubicin (Meda AB, Solna, Sweden) was used at 1 µM, gemcitabine (Eli Lilly Sweden AB, Solna, Sweden) at 200 µM, carboplatin (Teva Sweden AB, Helsingborg, Sweden) at 100 µM, bortezomib (Janssen-Cilag AB, High Wycombe, Buckinghamshire, UK) at 1.3 µM and pemetrexed (Eli Lilly Sweden AB) at 90 µM. The concentration of selenite (Na_2_SeO_3_, 10 µM, Sigma-Aldrich) was chosen on the basis of previous studies and expected tolerable doses *in vivo* [10], [11].

### Cell Cycle Analysis

The cells were grown as previously described and confluent cells were trypsinized, divided in two and reseeded in 75 cm^2^ flasks. After addition of fresh culture medium, cells were treated with either pemetrexed or PBS for the control cells. After 48 hours of treatment the culture medium was collected, cells were trypsinized and spun down. The pellet was then fixed in cold 70% ethanol. Samples were washed in PBS and resuspended in staining solution, containing 50 µg/ml propidium iodide (PI, Sigma-Aldrich) and 100 µg/ml ribonuclease A (Sigma-Aldrich) and incubated for 30 min at 37°C. The samples were then analyzed in a FACSCalibur cytometer (Becton Dickinson, Franklin Lakes, NJ, USA) and the CELLQuest Pro software. Live cells (gated based on Forward/Side Scatter (FSC/SSC) distribution in control cells) and cell cycle distribution was evaluated using FlowJo 7 for Windows (Tree Star Inc., Ashland, OR, USA). For each cell line, three experiments were performed and a representative experiment was chosen to demonstrate the effects on cell cycle in treated and untreated cells. PI intensity represents amount of DNA in live cells and % of max shows cells normalized according to FlowJo algorithms considering different amount of live cells in controls and treated cells.

### Immunocytochemistry

Cytospin of MM cells was performed on SuperFrost Plus glass slide (Thermo Fisher Scientific Inc, Waltham, MA, USA), fixed in ethanol/methanol containing 3% polyethylene glycol (PEG) and stored at –20°C. PEG was removed by treating slides with decreasing concentrations of ethanol. Immunostaining was performed in a Leica BOND-MAX automated IHC (see Table 1) with relevant isotype controls, diluted in BOND Primary Antibody Diluent (Leica Microsystems GmbH) and detected with the Bond Polymer Refine Detection kit (Leica Microsystems GmbH) according to the manufacturer’s protocol. Briefly, slides were pretreated 5 min in a citrate buffer pH 6.0 (Bond Epitope Retrieval Solution 1, Leica Microsystems GmbH) for all targets except for Pgp, where an EDTA buffer pH 9.0 (Bond Epitope Retrieval Solution 2, Leica Microsystems GmbH) was used. Endogenous peroxidase activity was abolished with hydrogen peroxide. Slides were then treated with primary antibodies for 30 min and a secondary IgG was added and incubated for 15 min. Following addition and incubation with a poly-HRP for 15 min, slides were incubated with Diaminobenzidine for 10 min and then treated with hematoxylin for 10 min. Slides were independently evaluated by two cytopathologists (KD and AH) who rated the staining intensity from 0 to 3 (0 representing no staining, 3 representing strong immunoreactivity).

**Table 1:** Antibodies used in these experiments.

Target	Abbreviation	Antibody	Dilution	Supplier	Product code
P-glycoprotein	Pgp	Mouse monoclonal	1∶10	1	NCL-PGLYm
Multidrug resistance-associated protein 1	MRP-1	Mouse monoclonal	1∶25	1	NCL-MRP1
Excision repair cross-complementing rodent repairdeficiency, complementation group 1	ERCC1	Mouse monoclonal Ab-2(clone 8F1)	1∶100	2	MS-671
Ribonucleotide reductase M1	RRM1	Rabbit polyclonal	1∶50	3	Ab81085
Thymidylate synthase	TS	Mouse monoclonal	1∶100	3	Ab58287
x_c_^-^ cystine transporter	xCT	Rabbit polyclonal	1∶1600	2	PA1-16893
20S proteasome	20S P	Rabbit polyclonal	1∶200	3	Ab22673

Suppliers: 1 = Leica Microsystems GmbH, Wetzlar, Germany, 2 = Thermo Fisher Scientific Inc, Waltham, MA, USA. 3 = Abcam, Cambridge, UK.

### Statistical Analyses

Results from cytotoxicity tests are mean values of at least three independent experiments, with four data points in each experiment. Synergistic and antagonistic effects of drug combinations were analyzed by defining the theoretical effect of a combination as the product of the observed effect of two single drugs after 48 hours of treatment. This was then compared to the observed effect at the same time point when combining the two drugs. Observed effects greater than the theoretical effects were defined as synergism and observed effects smaller than the theoretical effects as antagonism. To analyze significant effects within sub-groups, Wilcoxon signed-rank tests were performed. For all these analyses, statistical significance was accepted as p<0.05.

Results of cell cycle experiments are mean values of three independent experiments, where treated cells were normalized to untreated cells. Differences between the treated and control cells were analyzed using a one-tailed paired Student’s t-test.

To examine possible correlations of the immunoreactivity to the obtained drug cytotoxicity on the different cell lines, linear regression analyzes were performed. Similarly, correlations were performed comparing the proliferation, defined as the ratio between control cells after 24 and 48 hours, to the viability of cells treated with the respective drugs alone and in combination.

To analyze the effect of length/width ratio and immunoreactivity of predictive markers on viability we used multivariate regression. For each combination of drug and target protein, independent variables were: immunoreactivity, phenotype and their interaction, while viability was the dependent variable. Analysis of variance (ANOVA) was used to test statistical significance of each regression coefficient. Correction for multiple comparisons was performed across all fitted models using the False Discovery Rate (FDR) procedure. These analyzes were performed using R version 2.15.2 [47].

## Results

### Growth Characteristics of Mesothelioma Cell Lines

**Figure 1:**
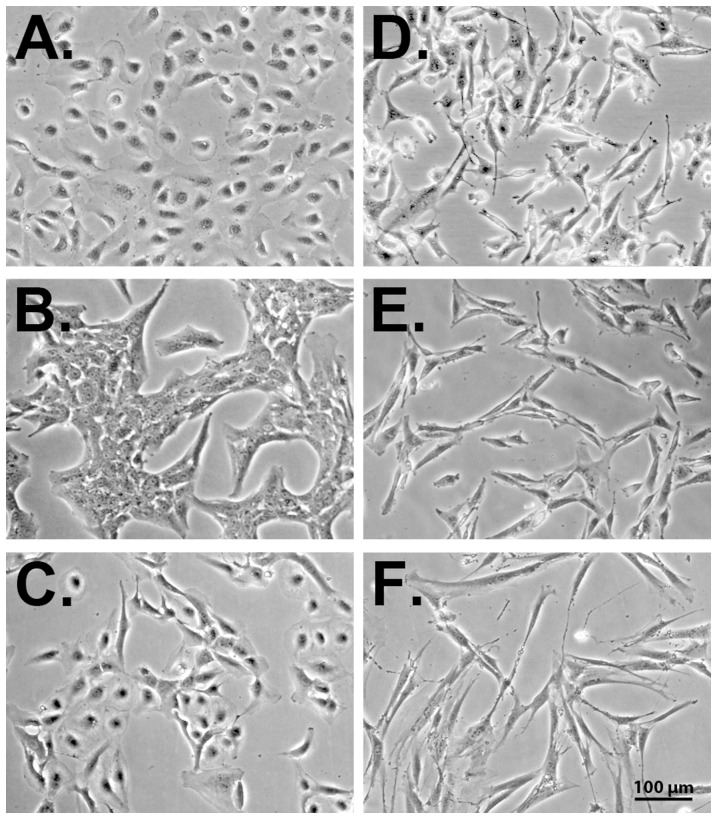
Morphological characteristics of the malignant mesothelioma cell lines. Characteristic micrographs presenting the different cell lines with increasing length/width ratios from A to F. A: Epitheliod STAV-AB cells. B: Biphasic M-14-K cells. C: Biphasic STAV-FCS cells. D: Biphasic ZL-34 cells. E: Biphasic JL-1 cells. F: Sarcomatoid DM-3 cells. Scale bar = 100 µm.

The examined mesothelioma cell lines showed variable growth patterns. STAV-AB showed polygonal cells, DM-3 displayed fibroblast-like cells and the remaining four cell lines exhibited a mixture of these two morphological types (Figure 1). This is reflected in the length/width ratios where STAV-AB cells had an average length/width ratio of 1.8, DM-3 cells an average ratio of 9.2 and the remaining four cell lines an average ratio between 2.6 and 3.8. Morphological heterogeneity in the four cell lines with mixed morphologies was reflected in large standard deviations on length/width ratios (Table 2). Based on these results, cell lines with an average length/width ratio below 2 were considered as epithelioid, between 2 and 4 as biphasic, and over 4 as sarcomatoid phenotypes.

**Table 2:** Characteristics of mesothelioma cell lines.

	Culture medium	Phenotype	Length/width	Doubling time	Established by
**STAV-AB**	**RPMI 1640 with 10% AB**	**Epithelioid**	**1.8±0.6**	**45 hours**	**Klominek J. ** [**34**]
M-14-K	RPMI 1640 with 5% FBS and 5% BS	Biphasic	2.6±1.3	55 hours	Pelin-Enlund K. [37]
STAV-FCS	RPMI 1640 with 5% FBS and 5% BS	Biphasic	3.2±2.0	90 hours	Klominek J. [34]
ZL-34	RPMI 1640 with 5% FBS and 5% BS	Biphasic	3.4±1.7	24 hours	Schmitter D. [35]
JL-1	NCTC-109 with 20% FBS	Biphasic	3.8±1.3	47 hours	Philippeaux MM. [36]
**DM-3**	**NCTC-109 with 20% FBS**	**Sarcomatoid**	**9.2±4.0**	**54 hours**	**Philippeaux MM. ** [**36**]

Length/width ratios are average with standard deviation. Epithelioid and sarcomatoid cell lines in bold. Abbreviations: RPMI 1640 = Gibco RPMI 1640 medium, AB = Human AB-serum, FBS = Fetal Bovine Serum, BS = Bovine Serum and NCTC-109 = NCTC-109 medium.

Doubling times of the included cell lines also varied considerably (Table 2). The ZL-34 cells were the fastest growing cells, doubling within 24 hours, while STAV-FCS cells were almost 4 times slower, doubling at 90 hours. The remaining four cell lines had doubling times ranging 45–55 hours.

### Cytotoxicity of Single Drug Treatment

Viability of treated cells was normalized to controls and effects were described as moderate (40–70% viable cells) or strong (<40% viable cells). Most of the examined mesothelioma cell lines were resistant to several drugs and only STAV-AB and ZL-34 cells were affected by more than three drugs.

**Figure 2:**
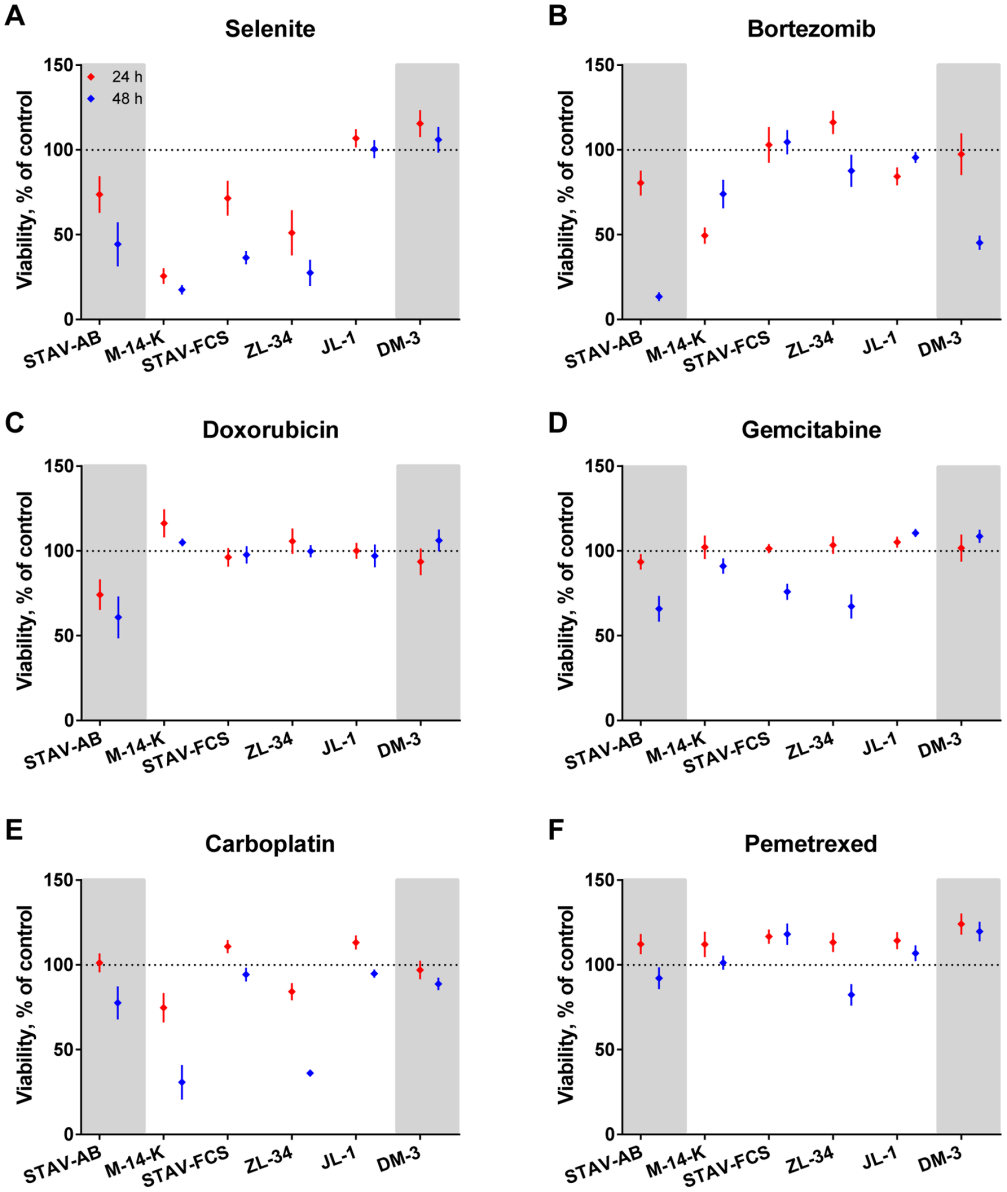
Sensitivity of mesothelioma cell lines to different cytotoxic drugs. Treated cells were normalized to untreated cells and cell viability was measured using the WST-1 assay after 24 (red) and 48 hours (blue). Results are mean values of at least three independent experiments with four replicates in each. Error bars denote the 95% confidence intervals. Cell lines are presented with increasing length/width ratios from left to right and divided into three sub-groups according to their phenotype, epithelioid cell line on the left and sarcomatoid to the right on grey background, biphasic in the middle.

The experimental drugs used in this study were more effective than the drugs conventionally used in clinics. Selenite showed strong to moderate effects on M-14-K, ZL-34, STAV-FCS and STAV-AB cells. However, cell lines with the highest length/width ratios, JL-1 and DM-3, were not affected (Figure 2A).

Bortezomib showed moderate to strong effects on DM-3 and STAV-AB cells (Figure 2B). M-14-K and JL-1 cells showed greater viability after 48 than 24 hours in contrast to the overall trend of time-dependently increasing cytotoxicity. Interestingly, biphasic cell lines were more resistant to bortezomib than the epitheloid and sarcomatoid cell lines.

Doxorubicin affected only the STAV-AB cells and in a moderate fashion (Figure 2C). Gemcitabine showed a moderate effect on STAV-AB and ZL-34 cells while carboplatin had a strong effect on M-14-K and ZL-34 cells (Figure 2D–E).

**Figure 3:**
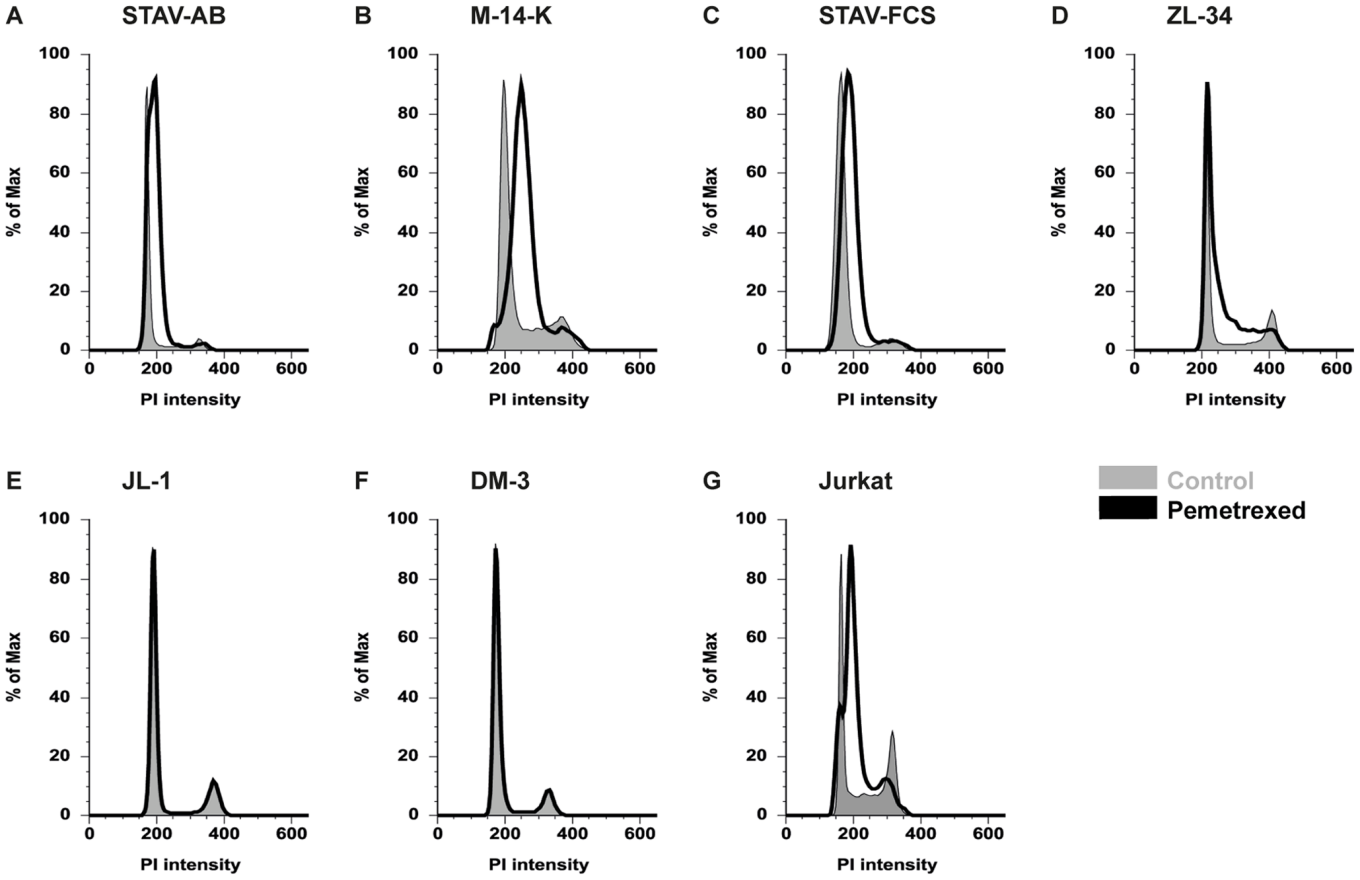
Cell cycle distribution after 48 hours of pemetrexed treatment. All cells were stained with PI. Cells were normalized according to FlowJo algorithms (% of max), considering the different amount of live cells in controls and treated cells. Mesothelioma cell lines arranged with increasing length/width ratios from A to F. One representative experiment is shown for each cell line with control cells marked in grey and 48 hours pemetrexed treated cells in black. STAV-AB (A), M-14-K (B), STAV-FCS (C) and Jurkat cells (G, used as positive control) show an S-phase arrest.

Pemetrexed was the least effective single drug when measured using WST-1 (Figure 2F). Jurkat cells, used as a positive control, showed strong effects (data not shown). To further investigate the cellular response to pemetrexed, we performed cell cycle analysis after 48 hours of treatment. We could see a statistically significant effect on the viability and the cell cycle distribution. The amount of live STAV-AB, Jurkat, M-14-K and STAV-FCS cells was significantly decreased (12–36%) after treatment (Table 3). In these cell lines we could also observe an increased PI intensity representing accumulation of cells in an early S-phase (Figure 3A–C and G). The increased PI intensity was to some extent also seen in ZL-34 cells, indicating an S-phase arrest in a subpopulation of cells (Figure 3D). JL-1 and DM-3 cells remained unaffected (Figure 3E–F).

**Table 3:** Proportion of live cells after 48 hours of pemetrexed treatment.

	% of control		p-value
**STAV-AB**	**64.4±21.8**	*	**0.01**
M-14-K	81.8±12.9	*	0.04
STAV-FCS	88.1±8.9	*	0.02
ZL-34	95.7±6.5		0.15
JL-1	98.2±2.2		0.18
**DM-3**	**101.3±2.8**		**0.27**
Jurkat	76.7±4.3	*	<0.001

Live cells after treatment, normalized to untreated control cells. Results are mean values from at least three independent experiments, with corresponding standard deviation. Mesothelioma cell lines arranged with increasing length/width ratios, with epithelioid and sarcomatoid cell lines in bold and Jurkat cells as positive control. Asterisks denote a significant differences between the treated and control cells using a paired student’s t-test with one-tailed p-values (p<0.05).

To see if the observed differences correlate to the proliferation rate of cells, untreated controls were plotted against the effects of the drugs at 48 hours (Figure S1). Weak but statistically significant inverse correlations were found for carboplatin (p = 0.007), pemetrexed (p = 0.008) and gemcitabine (p = 0.03), with R^2^-values of 0.14, 0.11 and 0.07, respectively. Thus, differences in proliferation rate only represented a minor factor in predicting the drug response.

### Combinations of Conventional Drugs

**Figure 4:**
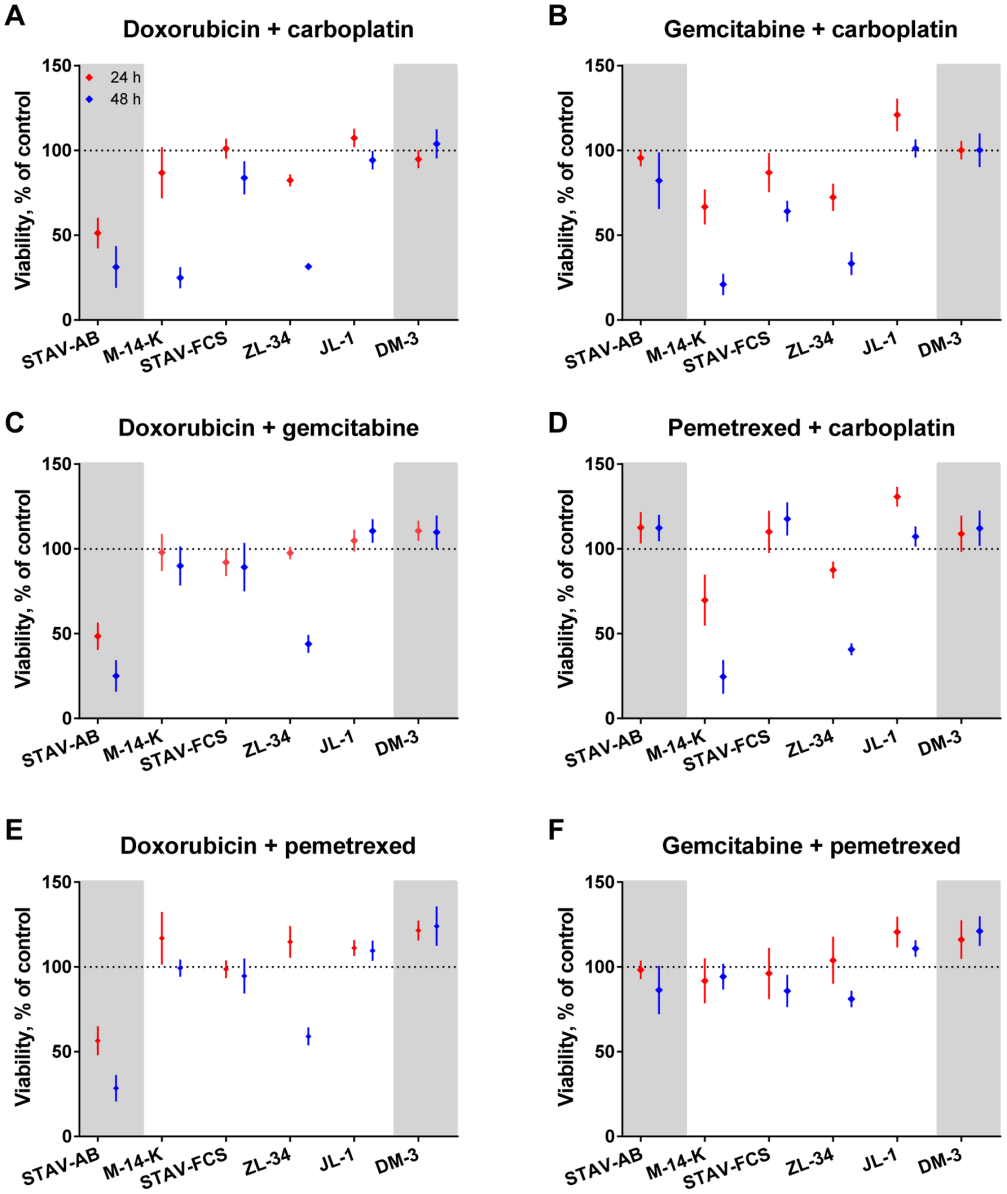
Effects of conventional drug combinations on mesothelioma cells. Cells were treated with combinations of the conventional drugs, normalized to untreated cells and viability was measured with the WST-1 assay after 24 (red) and 48 hours (blue). Mean values of at least three independent experiments with four replicates in each are presented. Error bars denote the 95% confidence intervals. Cell lines are divided into three sub-groups with increasing length/width ratios from left to right and according to their phenotype; biphasic in the middle, epithelioid cell line on the left and sarcomatoid to the right on grey background.

The above-described pattern of resistance against conventional drugs was also seen when these drugs were combined. Doxorubicin and carboplatin was the most effective conventional drug combination with strong effects on STAV-AB, M-14-K and ZL-34 cells (Figure 4A). Gemcitabine and carboplatin had moderate to strong effects on M-14-K, ZL-34 and STAV-FCS cells (Figure 4B).

The combination of doxorubicin and gemcitabine showed a strong to moderate effect on STAV-AB and ZL-34 cells (Figure 4C). Pemetrexed and carboplatin had a strong effect on M-14-K cells and ZL-34 cells were moderately affected (Figure 4D). Doxorubicin and pemetrexed showed strong effects on STAV-AB cells and ZL-34 cells were moderately affected by this drug combination (Figure 4E). The combination of gemcitabine and pemetrexed was the least effective combination (Figure 4F).

### Drug Combinations with Selenite were Most Effective Against the Mesothelioma Cells

**Figure 5:**
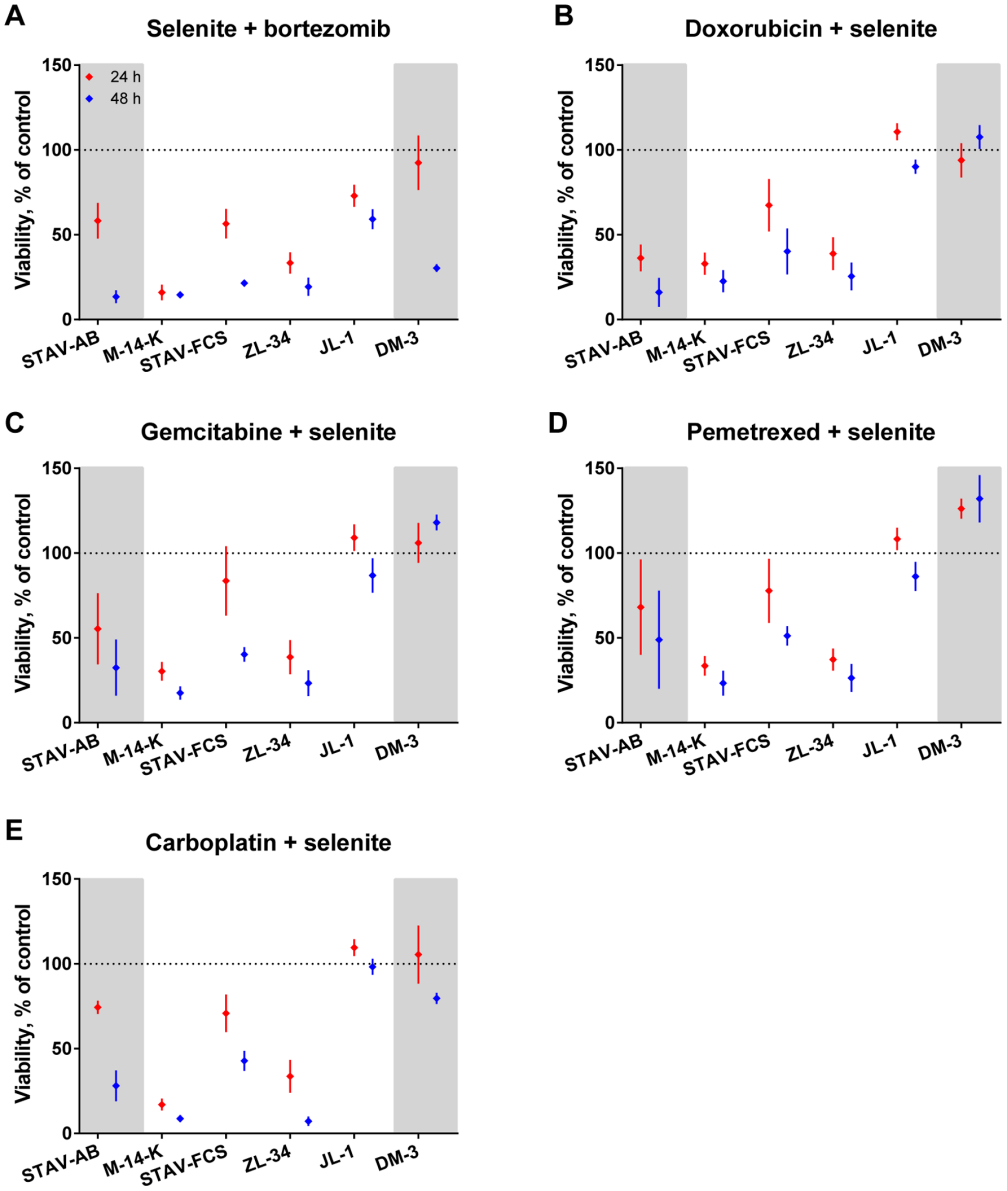
Drug combinations with selenite, cytotoxic effects on mesothelioma cells. Cells exposed to selenite in combination with the remaining five drugs, normalized to untreated cells. Viability was measured with the WST-1 assay after 24 (red) and 48 hours (blue). The six different cell lines are shown with increasing length/width ratios and in three phenotypic sub-groups; biphasic cell lines in the middle, epithelioid on the left and sarcomatoid to the right on grey background. Results are mean values of at least three independent experiments with four replicates in each. Error bars denote the 95% confidence intervals.

Drug combinations including selenite were cytotoxic to all six cell lines included in this study (Figure 5). Selenite and bortezomib was the most cytotoxic drug combination in this study (Figure 5A). After 24 hours of treatment, the combination showed a strong to moderate effect on M-14-K, ZL-34, STAV-FCS and STAV-AB cells. This was increased after 48 hours of treatment and after this time also DM-3 and JL-1 cells were affected in a strong to moderate fashion.

The combination of selenite and doxorubicin, gemcitabine, pemetrexed or carboplatin affected the cell lines in a similar manner (Figure 5B–E). JL-1 and DM-3, the two cell lines with the highest length/width ratios, were unaffected by these combinations at both time points.

### Drug Combinations with Bortezomib had a Strong Effect on a Subset of Mesothelioma Cell Lines

**Figure 6:**
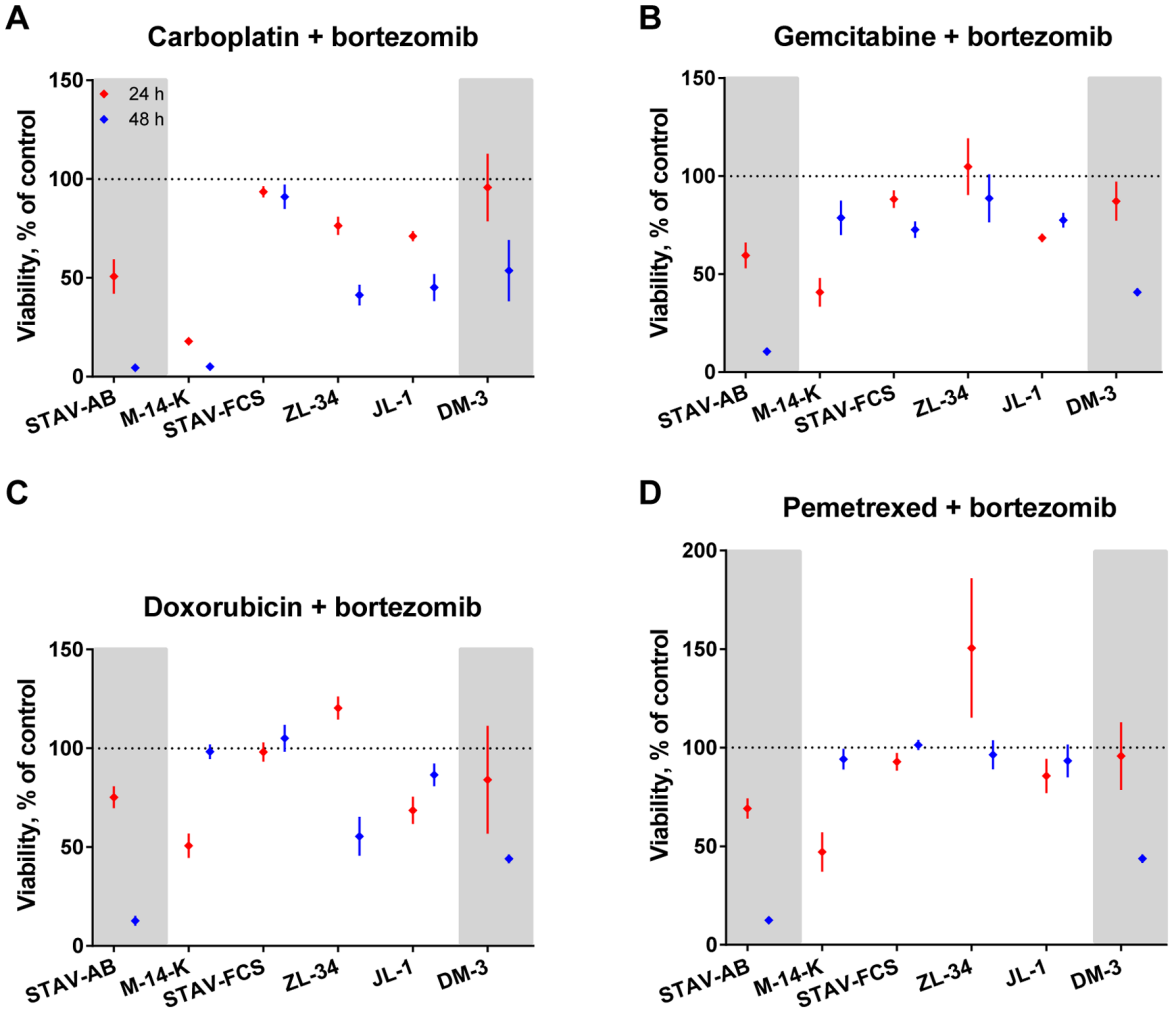
Effects of drug combinations with bortezomib on mesothelioma. Viability of cells measured using the WST-1 assay after 24 (red) and 48 hours (blue) of treatment with bortezomib in combination with the four conventional drugs and normalized to untreated cells. Results are shown as mean values of at least three independent experiments with four replicates in each experiment. Error bars denote the 95% confidence intervals. Cell lines are presented with increasing length/width ratios from left to right and divided into three sub-groups according to their phenotype, epithelioid cell line on the left and sarcomatoid to the right on grey background, biphasic in the middle.

The drug combinations with bortezomib strongly affected some of the cell lines. In three of the drug combinations, the effect was greatest at 24 hours and the cells then recovered after 48 hours. This was observed in M-14-K and JL-1 (Figure 6B–D). The combination of carboplatin and bortezomib had strong effects on M-14-K and STAV-AB cells while ZL-34, JL-1 and DM-3 cells were moderately affected (Figure 6A).

Bortezomib and gemcitabine strongly affected STAV-AB cells and moderately affected DM-3 cells (Figure 6B). There was a moderate effect on M-14-K and JL-1 cells after 24 hours of treatment and contrary to the overall trend of time-dependent effect, the two cell lines showed a greater viability after 48 hours than after 24 hours.

The combination of doxorubicin and bortezomib had strong influence on STAV-AB cells (Figure 6C). DM-3 and ZL-34 cells were moderately affected after 48 hours and similar to the combination of bortezomib and gemcitabine the JL-1 and M-14-K cells had a higher viability after 48 hours than after 24 hours of doxorubicin and bortezomib treatment.

Pemetrexed and bortezomib had a moderate influence on M-14-K and STAV-AB cells after 24 hours of treatment and these effects were potentiated in STAV-AB cell after 48 hours, whereas M-14-K cells recovered after 48 hours (Figure 6D). The DM-3 cells were moderately affected.

### Drug Combinations Showed no Major Synergistic or Antagonistic Effects

**Figure 7:**
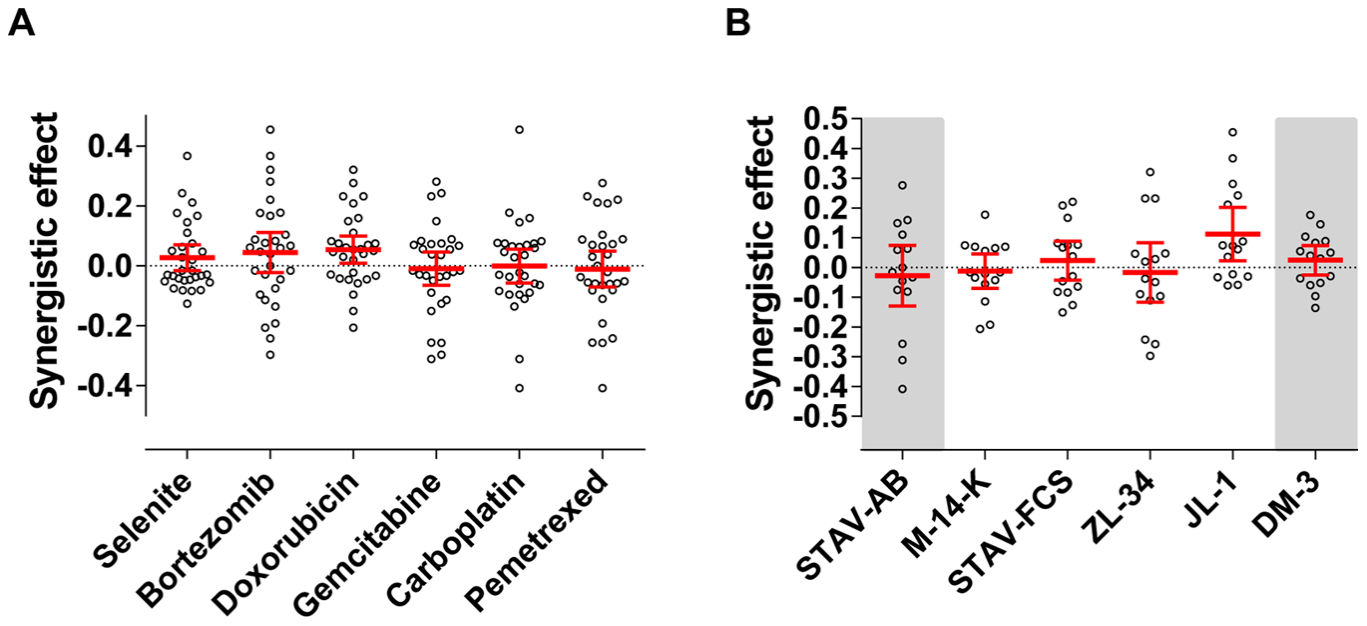
Synergistic and antagonistic effects of drug combinations. Comparison between mean effects of drug combinations and the theoretical effects, defined as the product of the mean effects of the two single drugs in the combination. Each data point represents a comparison and error bars in red denote the 95% confidence intervals. Statistical significance was accepted as p<0.05. A: Synergistic and antagonistic effects with respect to the different drugs. Doxorubicin was significantly different from zero (Wilcoxon signed-rank test). B: Synergistic and antagonistic effects with respect to the different cell lines. Cell lines are arranged with increasing length/width ratios from left to right and divided into three sub-groups according to their phenotype, epithelioid cell line on the left and sarcomatoid to the right on gray background, biphasic in the middle. Subpopulations within the cell lines can be distinguished in JL-1, STAV-AB and ZL-34 cells. Wilcoxon signed-rank test showed that JL-1 were significantly different from zero.

In general, neither major synergistic nor antagonistic patterns could be demonstrated, even though some individual effects were significantly different from zero. Doxorubicin was the only drug yielding statistically significant synergism in combination with other drugs and JL-1 was the only cell line where a significant pattern of synergistic effects from drug combinations could be observed (Figure 7A–B). The largest synergistic effects were found when treating JL-1 cells with a combination of bortezomib with carboplatin, selenite or gemcitabine. Interestingly, the combination of pemetrexed and carboplatin showed substantial antagonistic effects on STAV-AB cells.

### MRP-1 and xCT Immunoreactivity Predicted Carboplatin and Pemetrexed Effect

**Figure 8:**
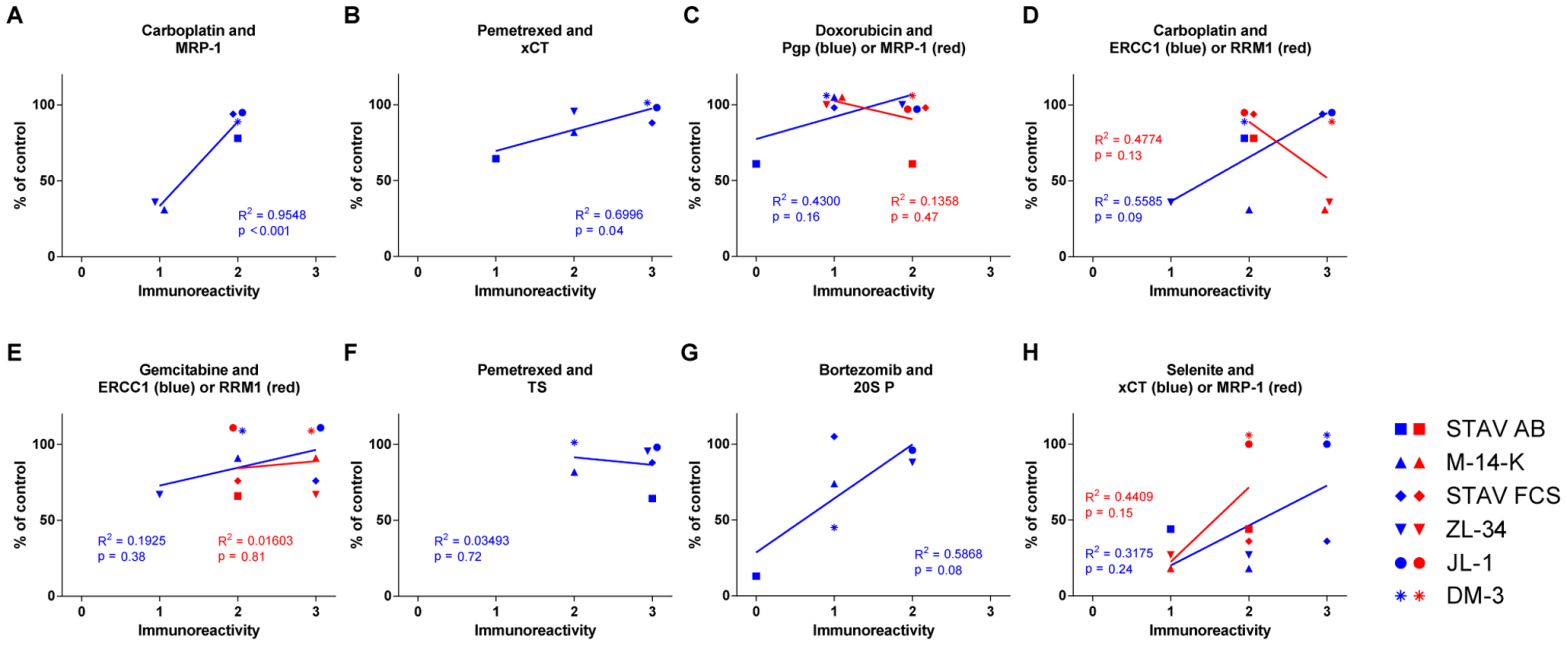
Correlation between immunoreactivity of predictive markers and drug sensitivity. The sensitivity of mesothelioma cell lines to different drugs plotted against the immunoreactivity of different predictive markers, presented together with results from the linear regression analyses. Statistical significance was accepted at p<0.05 and was seen for MRP-1 immunoreactivity and sensitivity of cell lines to carboplatin treatment (A) and for xCT and pemetrexed effect (B). Abbreviations: Pgp = P-glycoprotein, MRP-1 = Multidrug resistance-associated protein 1, ERCC1 = Excision repair cross-complementing rodent repair deficiency, complementation group 1, RRM1 = Ribonucleotide reductase M1, TS = Thymidylate synthase, xCT = x_c_^-^ cystine transporter and 20S P = 20S proteasome.

Immunoreactivity of the different predictive markers varied between cell lines and when plotted against drug sensitivity none of the hypothesized correlations were observed. Unexpectedly, immunoreactivity of MRP-1 in the cellular cytoplasm, significantly predicted the sensitivity of the cell lines to treatment with carboplatin (p<0.001), with a R^2^-value of 0.95 (Figure 8A). Similarly, membrane staining for xCT significantly predicted pemetrexed effect (p = 0.04) in cell cycle analysis but with a lower R^2^-value (0.70, Figure 8B). When correlating doxorubicin cytotoxicity and MRP-1 staining intensity and also carboplatin effect and RRM1 cytoplasm staining, we found inverse but not significant correlations (Figure 8C-D). Pgp was not detected in membranes of STAV-AB cells and was not correlated to cytotoxicity of doxorubicin (Figure 8C). Nuclear ERCC1 and cytoplasmic RRM1 could be detected in all cell lines but did not predict sensitivity to gemcitabine treatment (Figure 8E). TS had a strong immunoreactivity in the cytoplasm and nucleus of each cell line but no correlation to pemetrexed effect was detected (Figure 8F). When plotting carboplatin effect and ERCC1 staining intensity as well as bortezomib effect and 20S P staining in cytoplasm and nucleus, no significant correlations were found with R^2^-values of 0.56 and 0.59 (Figure 8D and G). Membrane staining for xCT and cytoplasmic staining for MRP-1 did not correlate to selenite effect (Figure 8H).

### Phenotype and ERCC1 Immunoreactivity Together Predicted Outcome of Gemcitabine Treatment

Combining length/width ratio and immunoreactivity of studied markers predicted the effect of several drugs. ERCC1 and phenotype was strongly correlated to the sensitivity of cell lines to gemcitabine treatment (Table 4, p<0.0001). The cytotoxicity of doxorubicin was inversely correlated to the interaction of Pgp staining and length/width ratio. RRM1 and phenotype had an inverse correlation to gemcitabine cytotoxicity. Similar correlations were observed for bortezomib and 20S P. Correlation for the interaction of phenotype and xCT was found for the effect of selenite or pemetrexed. When summarizing all coefficients we could see that the impact of marker reactivity, phenotype and the interaction of these was variable (Table S1). The largest regression coefficient and was found for the phenotype effect in the ERCC1 and selenite combination.

**Table 4:** Correlation of predictive markers and phenotype interactions to drug sensitivity.

Drug	Predictive marker	Interaction coefficients	p
Selenite	xCT	25.21	<0.001
	MRP-1	−3.98	0.73
Bortezomib	20S P	−29.47	<0.001
Doxorubicin	Pgp	−23.15	<0.001
	MRP-1	11.30	0.20
Gemcitabine	ERCC1	51.79	<0.001
	RRM1	−13.93	<0.001
Carboplatin	ERCC1	−2.55	0.86
	RRM1	−0.80	0.79
	MRP-1	−5.90	0.47
Pemetrexed	TS	3.02	0.21
	xCT	11.73	<0.001

Interaction coefficients and p-values from the multivariate regression and ANOVA analysis for the suggested predictive markers. Gemcitabine and ERCC1 present the largest coefficients.

## Discussion

Current treatment strategies for malignant mesothelioma have limited effect and there is a great need for improved treatment. The best-performing regime combines pemetrexed and cisplatin, yielding response rates of 40% at best [14]. Since response rates are correlated to tumor phenotype and overall survival [4], [48], it is important to find predictive markers that identify responders and non-responders before treatment is initiated. Together with new drugs and better drug combinations, as well as an improved understanding of the differences in response between patients, the outcome of MM patients can hopefully be improved.

In this study, the two experimental drugs, selenite and bortezomib, were more effective than the panel of conventionally used cytotoxic drugs. In our experimental setup, selenite was the most effective single drug, affecting the epithelioid cell line and three out of four biphasic cell lines (Figure 2A). The two cell lines with the highest length/width ratio remained unaffected, in contrast to our previous results in which we observed a phenotype-dependent effect with greater effects in sarcomatoid cells [10], [11]. Drug combinations with selenite demonstrated a strong cytotoxicity that was further increased by bortezomib (Figure 5A), and this was the most powerful drug combination in this study.

Selenite exerts its cytotoxic effect mainly by oxidizing free thiols, generating reactive oxygen species intracellularly and inducing oxidative stress in malignant cells [10], [49], [50]. This effect is decreased when adding antioxidants [10], [51], [52], [53], [54] and in cells with an induced higher expression of antioxidant proteins [55]. Also, selenite cytotoxicity is dependent on its uptake by the cell. No specific protein responsible for the internalization of selenite has been found, but high expression of xCT and MRP-1, increases cellular uptake of cystine and secretion of cysteine. Cysteine reduces selenite extracellularly which then results in a higher uptake of cystine by the cell [31], [32], presumably leading to a tumor specific response. Surprisingly, in our studies the immunoreactivity of MRP-1 and xCT did not predict the effect of selenite on the used mesothelioma cell lines.

In patients, radioactive selenite has been shown to selectively accumulate in malignant tissue [56], probably due to the hypoxic and reducing extracellular environment in many malignant tissues, causing an increased reduction of selenite and thus an increased uptake. Adding selenite to standard treatment of patients with different malignancies has been shown to reduce side effects of treatment [17], [18], [57].

Bortezomib affected the epithelioid and sarcomatoid cell lines as well as one of the biphasic cell lines (Figure 2B). When combining the experimental drug with conventional drugs, the effect was potentiated and it was strongest with carboplatin (Figure 6A). Bortezomib has previously been demonstrated to have strong cytotoxic effects on adherent mesothelioma cells [19], [20], [58] and spheroids [59], [60]. However, in a phase II clinical trial, performed with MM patients treated with bortezomib as a single agent the outcome was insufficient [61]. This was in contrast to earlier studies performed in patients with multiple myeloma [62], [63], were the effect of bortezomib was further increased by adding doxorubicin to treatment [64]. In primary cells from patients with acute myeloid leukemia it has been shown that cells with high levels of 20S P are more sensitive to treatment with bortezomib [33]. Bortezomib affects cells by binding the 20S P and causes an inhibition of the proteasome function in healthy and malignant cells [65], [66], [67], [68], [69]. However, malignant cells have a higher proteasome expression and activity making them more sensitive to proteasome inhibition [70], [71], [72]. The proteasome degrades intracellular proteins, such as cyclins, caspases and nuclear factor κB, proteins regulating cellular proliferation and apoptosis. Malignant cells often acquire mutations in proteins involved in these pathways during tumorigenesis and inhibition of the proteasome might overcome some of these effects, as reviewed in [73]. The impact of bortezomib on the cell lines used in these experiments was not predicted by the immunoreactivity of 20S P but through the interaction of phenotype and immunoreactivity (Figure 8G and Table 4).

Selenite and bortezomib target different molecular pathways than the conventional drugs, and this might partly explain why they outperformed the conventional drugs. Doxorubicin, gemcitabine and carboplatin were generally ineffective on the examined cell lines. This may reflect the characteristic drug resistance of mesothelioma cells but it also may indicate that there are differences between the efficacy of drugs on cell lines and on tumors *in vivo*.

Surprisingly, pemetrexed did not affect the survival of any cell lines used in the cytotoxicity tests (Figure 2F), even though the drug is used as standard treatment. The drug however showed increased cytotoxicity in combination with doxorubicin (Figure 4E) although antagonistic effects were observed with carboplatin on STAV-AB cells (Figure 7A-B). In contrast to the cytotoxicity tests, effect of pemetrexed was extensive in the three cell lines with the most epithelioid phenotype in the cell cycle analysis. A significant decrease of the living cell population was seen and in remaining live cells a substantial S-phase arrest was observed (Table 3 and Figure 3A-C), in concordance with previously reported data [74], [75], [76], [77]. The variability between the effects of pemetrexed in the two experimental settings can be explained by measurement of different cellular responses. We suspect that the response is complex and, apart from cell cycle arrest, the drug increases metabolic activity concealing cytotoxic effects. This could explain the WST-1 results. Also, further limitations of the WST-1 assay have previously been reported [78]. These observations highlight the need to study cell viability using several methods. Importantly, cell cycle analysis seems to reflect the effects of pemetrexed observed in clinical settings.

TS, the proposed predictive marker for pemetrexed sensitivity could not be correlated to the sensitivity of MM cells, while a significant correlation to xCT reactivity was found (Figure 8B and F and Table 4). This correlation, to our knowledge, has not been previously reported.

The combination of doxorubicin, gemcitabine and carboplatin has shown to be effective in a clinical trial [16]. In this study, the effects of these three drugs used as single agent were limited but in the combinations, significant synergistic effects were induced by doxorubicin (Figure 7A). Carboplatin or gemcitabine effect was not correlated to the immunoreactivity of proposed sensitivity markers ERCC1 and RRM1 (Figure 8D-E). However, the effect of carboplatin correlated to MRP-1 reactivity (Figure 8A). MRP-1 is frequently expressed in cells from mesothelioma patients [79] but the correlation to carboplatin sensitivity, to our knowledge, has not been previously reported. Multivariate regression analyses showed significant interactions of the target proteins and phenotype for gemcitabine and ERCC1 or RRM1 and for doxorubicin and Pgp (Table 4).

The effect of the different drugs and drug combinations in this study was more prominent on the cell lines with lower length/width ratios, except for the effect of bortezomib, were a higher cytotoxicity was seen in the sarcomatoid cells then in biphasic cells. This is in line with findings that patients with a tumor dominated by sarcomatoid cells have a worse prognosis [4] but also highlights the need for more individualized treatment of patients with MM and a development of methods to predict the drug sensitivity of each individual patient.

### Conclusions

In this study the two experimental drugs, selenite and bortezomib, showed superior effect compared to conventional drugs. This motivates clinical testing of these agents as future treatment regime components for patients with MM. We demonstrate an extensive S-phase arrest in pemetrexed-sensitive cell lines but not decreased metabolic activity. This pinpoints the need to study cell viability using several methods.

Generally, proposed predictive markers failed to foresee sensitivity of cell lines in this study. Some unexpected correlations were however found. Thus, immunoreactivity of MRP-1 significantly predicted sensitivity of cell lines to treatment with carboplatin and reactivity of xCT significantly predicted pemetrexed effect. Impact of predictive markers might be increased by measuring several of them simultaneously. Predicting the outcome of patients by combining several markers has previously been achieved when looking at gene expression in surgically treated patients and immunostaining in patients treated with cisplatin and vinorelbine [80], [81].

These results indicate a possible use of drug sensitivity tests and combinations of predictive markers prior to the choice of therapy in each individual case. Such studies could be performed with MM cells obtained from effusions and are in progress in our laboratory.

All together these results present a broad variability between mesothelioma cell lines, with phenotypic and individual differences in drug sensitivity and reactivity of predictive markers. This demonstrates a need for more individualized treatment of patients with MM based on the sensitivity of individual tumors. We hypothesize that the optimal treatment strategy for each individual patient with malignant mesothelioma might be predicted by studying the sensitivity/resistance profile of their primary tumor cells to a panel of cytotoxic drugs.

## Supporting Information

Figure S1 **Correlation of drug sensitivity to proliferation rate of cells.** Effects of single drugs at 48 hours on the six different mesothelioma cell lines are plotted against the proliferation rate of untreated control cells. Regression lines for each cell line are shown in red. Statistically significant departure of the slope from 0 was accepted at p<0.05. D-F: Significant correlations between drug effect and cell proliferation can be seen but with a very low explanatory value (R^2^).
(TIF)

Table S1 **Regression coefficients from multivariate regression models.** A. Correlation of predictive markers and drug sensitivity. B. Correlation of length/width ratio and drug sensitivity. C. Correlation of predictive markers and length/width ratio interactions with drug sensitivity: Effect of the respective independent variable on viability, as estimated by regression coefficient. A: Phenotype. ERCC1, selenite and bortezomib display the largest explanatory effects on the drug sensitivity. B: Predictive markers. The largest coefficient was found for ERCC1 and selenite. C: Effect of interaction. ERCC1, selenite and bortezomib present the largest regression coefficients.
(DOC)
