# Supplementary figures and images for: Variation in Drug Sensitivity of Malignant Mesothelioma Cell Lines with Substantial Effects of Selenite and Bortezomib, Highlights Need for Individualized Therapy
Source: PLoS One. 2013 Jun 20;8(6):e65903. doi: 10.1371/journal.pone.0065903 (PMC3688685; doi:10.1371/journal.pone.0065903)

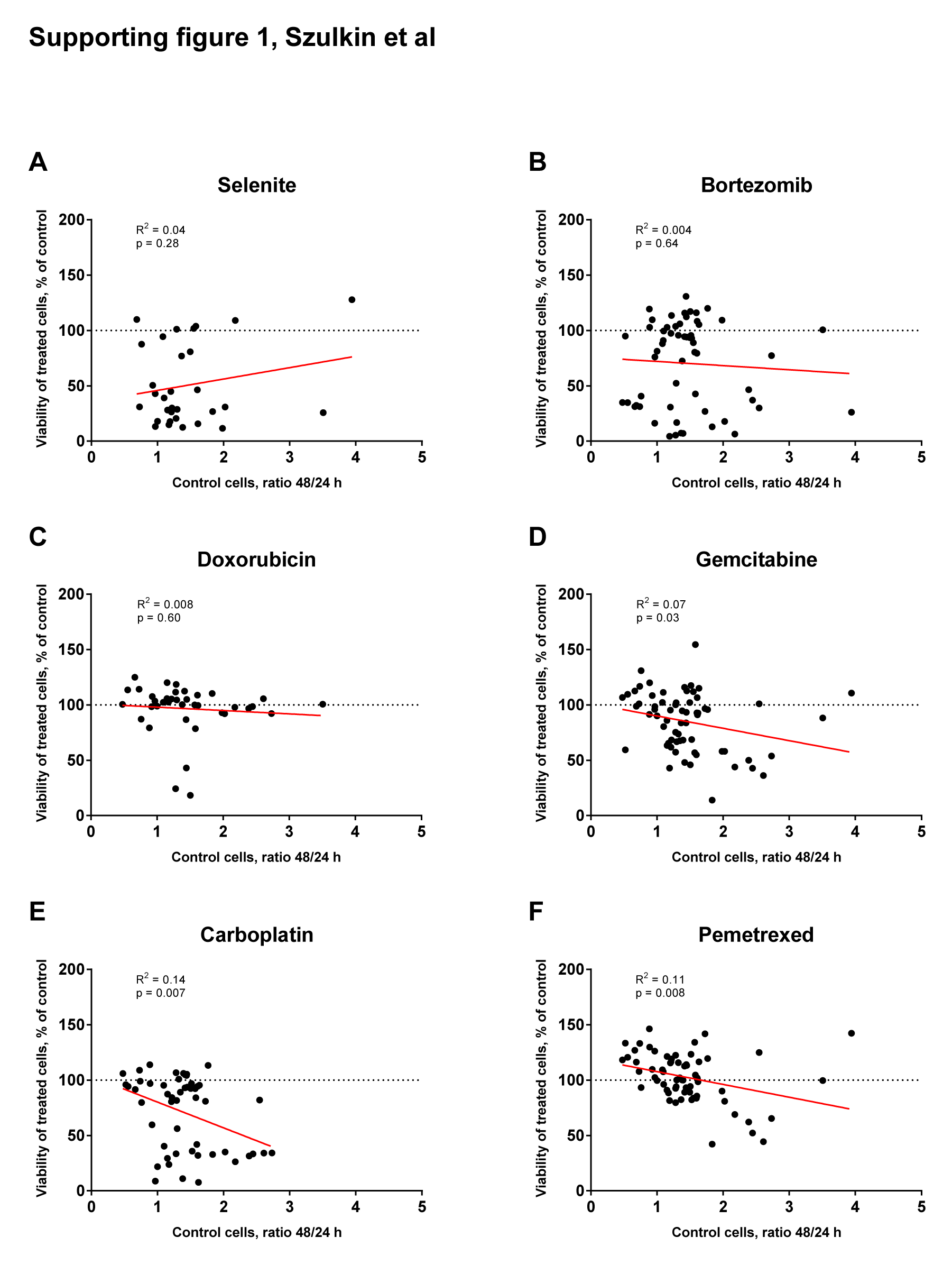

Supplement: Figure S1 — Correlation of drug sensitivity to proliferation rate of cells. Effects of single drugs at 48 hours on the six different mesothelioma cell lines are plotted against the proliferation rate of untreated control cells. Regression lines for each cell line are shown in red. Statistically significant departure of the slope from 0 was accepted at p<0.05. D-F: Significant correlations between drug effect and cell proliferation can be seen but with a very low explanatory value (R2). (TIF) [file pone.0065903.s001.tif]
