# Supplementary material for: Variation in Drug Sensitivity of Malignant Mesothelioma Cell Lines with Substantial Effects of Selenite and Bortezomib, Highlights Need for Individualized Therapy
Source: PLoS One. 2013 Jun 20;8(6):e65903. doi: 10.1371/journal.pone.0065903 (PMC3688685; doi:10.1371/journal.pone.0065903)
Supplement: Table S1 — Regression coefficients from multivariate regression models. A. Correlation of predictive markers and drug sensitivity. B. Correlation of length/width ratio and drug sensitivity. C. Correlation of predictive markers and length/width ratio interactions with drug sensitivity: Effect of the respective independent variable on viability, as estimated by regression coefficient. A: Phenotype. ERCC1, selenite and bortezomib display the largest explanatory effects on the drug sensitivity. B: Predictive markers. The largest coefficient was found for ERCC1 and selenite. C: Effect of interaction. ERCC1, selenite and bortezomib present the largest regression coefficients. (DOC) [file pone.0065903.s002.doc]

Supporting table 1. R**egression coefficients from multivariate regression models.**

A. Correlation of predictive markers and drug sensitivity

|  | Selenite | Bortezomib | Doxorubicin | Gemcitabine | Carboplatin | Pemetrexed |
| --- | --- | --- | --- | --- | --- | --- |
| MRP-1 | 48.60 | 42.69 | -54.53 | -103.51 | 70.57 | -66.82 |
| ERCC1 | -310.46 | -112.29 | 22.49 | -166.86 | 38.30 | 92.43 |
| RRM1 | -15.95 | 159.66 | 76.56 | 37.20 | -53.69 | 4.26 |
| TS | 24.48 | -139.93 | -80.30 | -48.16 | 67.93 | -10.82 |
| xCT | -40.69 | 94.19 | 46.71 | 0.54 | -60.53 | -11.06 |
| 20S P | -124.17 | 121.06 | 80.62 | -1.82 | -66.66 | 33.49 |
| Pgp | -124.17 | 121.06 | 80.62 | -1.82 | -66.66 | 33.49 |

B. Correlation of length/width ratio and drug sensitivity

|  | Selenite | Bortezomib | Doxorubicin | Gemcitabine | Carboplatin | Pemetrexed |
| --- | --- | --- | --- | --- | --- | --- |
| MRP-1 | 16.44 | 34.37 | -17.78 | -64.73 | 12.67 | -50.21 |
| ERCC1 | -181.10 | -68.80 | 15.04 | -98.55 | 8.87 | 48.66 |
| RRM1 | 37.21 | 136.36 | 61.04 | 46.15 | 11.26 | 22.93 |
| TS | 9.56 | -96.00 | -42.04 | -20.84 | 25.04 | -3.25 |
| xCT | -67.47 | 62.41 | 49.86 | -11.80 | -115.02 | -34.47 |
| 20S P | -31.17 | 24.39 | 23.93 | 1.76 | -14.82 | 15.07 |
| Pgp | -31.17 | 24.39 | 23.93 | 1.76 | -14.82 | 15.07 |

C. Correlation of predictive markers and length/width ratio interactions with drug sensitivity

|  | Selenite | Bortezomib | Doxorubicin | Gemcitabine | Carboplatin | Pemetrexed |
| --- | --- | --- | --- | --- | --- | --- |
| MRP-1 | -3.98 | -17.29 | 11.30 | 34.99 | -5.90 | 26.53 |
| ERCC1 | 95.61 | 33.92 | -5.66 | 51.79 | -2.55 | -22.58 |
| RRM1 | -7.92 | -47.57 | -20.20 | -13.93 | -0.80 | -6.25 |
| TS | 1.92 | 45.82 | 21.11 | 11.75 | -8.12 | 3.02 |
| xCT | 25.21 | -23.97 | -16.29 | 4.93 | 38.33 | 11.73 |
| 20S P | 45.11 | -29.47 | -23.15 | 3.16 | 20.54 | -12.83 |
| Pgp | 45.11 | -29.47 | -23.15 | 3.16 | 20.54 | -12.83 |

Effect of the respective independent variable on viability, as estimated by regression coefficient. A: Phenotype. ERCC1, selenite and bortezomib display the largest explanatory effects on the drug sensitivity. B: Predictive markers. The largest coefficient was found for ERCC1 and selenite. C: Effect of interaction. ERCC1, selenite and bortezomib present the largest regression coefficients.
